# Beneficial Effects of Melatonin on Periodontitis Management: Far More Than Oral Cavity

**DOI:** 10.3390/ijms232314541

**Published:** 2022-11-22

**Authors:** Chuan Wang, Leilei Wang, Xiaoxuan Wang, Zhengguo Cao

**Affiliations:** 1 The State Key Laboratory Breeding Base of Basic Science of Stomatology (Hubei-MOST KLOS) & Key Laboratory of Oral Biomedicine Ministry of Education (KLOBME), School & Hospital of Stomatology, Wuhan University, Wuhan 430079, China; 2 Department of Periodontology, School & Hospital of Stomatology, Wuhan University, Wuhan 430079, China; 3 Division of Oral and Maxillofacial Surgery, Faculty of Dentistry, The University of Hong Kong, Hong Kong SAR, China

**Keywords:** periodontitis, tissue destruction, systemic comorbidities, melatonin, adjunctive treatment, host-modulation therapy

## Abstract

Periodontitis as a highly prevalent chronic infection/inflammatory disease can eventually lead to tooth loss and masticatory dysfunction. It also has a negative impact on general health and largely impairs quality of life. The tissue destruction during periodontitis is mainly caused by the excessive immune–inflammatory response; hence, how to modulate the host’s reaction is of profound importance for effective periodontal treatment and tissue protection. Melatonin, as an endogenous hormone exhibiting multiple biological functions such as circadian rhythm regulation, antioxidant, and anti-inflammation, has been widely used in general healthcare. Notably, the past few years have witnessed increasing evidence for the application of melatonin as an adjunctive approach in the treatment of periodontitis and periodontitis-related systemic comorbidities. The detailed underlying mechanisms and more verification from clinical practice are still lacking, however, and further investigations are highly required. Importantly, it is essential to establish standard guidelines in the near future for the clinical administration of melatonin for periodontal health and general wellbeing.

## 1. Introduction

Periodontitis is a bacteria-induced, chronic infection/inflammatory disease characterized by progressive destruction of tooth-supporting tissues. Periodontitis has become the main reason for tooth loss/edentulism in adults worldwide [1]. It is also linked closely with other systemic diseases such as cardiovascular disease, Alzheimer’s disease, diabetes, and cancer, thus profoundly impairing people’s quality of life [2,3]. As the most common chronic inflammatory disease of humans, periodontitis has brought about huge socioeconomic impacts and healthcare costs [4]. Of note, the prevention and treatment of periodontitis have become the priority for periodontal research and clinical practice.

The occurrence of periodontitis is due to microbial dysbiosis and dysregulated host response. The accumulation of a dental plaque biofilm initiates the local inflammation (gingivitis), which in turn accelerates the dysbiotic environment and leads to dysregulation of the host immune–inflammatory response. Excessive release of inflammatory cytokines and chemokines, enhanced reactive oxygen species (ROS), and imbalanced bone metabolism further result in the destruction of periodontal tissues [5]. Herein, the ultimate objective of periodontal treatment is to modulate the excessive immune–inflammatory response and to rebuild the symbiotic environment between microbes and the host.

Currently, mechanical plaque removal by scaling and root planing (SRP) is the most widely applied method for the treatment of periodontitis [6]. Periodontal status in most patients could be improved after the performance of these basic periodontal therapies. However, simple plaque removal cannot totally quench the excessive immune–inflammatory response and re-establish the imbalanced microenvironment; progressive attachment loss still exists in certain patients after SRP [7]. Moreover, the degree of periodontal tissue destruction and the reaction to periodontal therapy vary greatly among individuals due to the discrepancy of host’s genetic risk factors and systemic conditions, together with the environmental and acquired risk factors. Thereby, adjunctive treatment such as host-modulation therapy might be a better choice that should be considered.

The 2017 Nobel Prize in Physiology or Medicine was awarded for ‘the discoveries of molecular mechanisms controlling the circadian rhythm’ [8]. Melatonin, an endogenous hormone that controls the sleep–wake cycle, began to draw people’s attention due to its multiple biological effects. Numerous functions of melatonin, such as circadian rhythm regulation, anti-infection, anti-inflammation, antioxidant, bone remodeling, etc., have been identified. Clinical studies mainly focus on its therapeutic effects on sleep and circadian disorders, neuroprotection, cancer, and immunological applications [9]. Of note, growing attention has focused on its utilization in the field of periodontology as a host-modulation agent, with positive conclusions obtained from both laboratory work and clinical trials [10,11,12,13], although there is still no standard protocol for its precise administration in clinical practice. More investigations are required before its wide application.

The aim of this review is to summarize and discuss current evidence for the application of melatonin in periodontal treatment, including its beneficial effects on periodontal parameters, and most importantly, on periodontitis-related systemic comorbidities. First, however, a brief introduction is given on the importance and the pathogenesis of periodontitis, and the limitation of current treatment methods, to provide a better understanding of the necessity and advantage for using melatonin as an adjunctive approach for the treatment of periodontitis.

## 2. Periodontitis: Importance, Pathogenesis, and Treatment

### 2.1. Importance of Periodontitis: ‘Local’ Lesion with Huge Disasters

Periodontitis is a chronic multifactorial inflammatory lesion that gradually destroys periodontium, which contains hard tissues such as alveolar bone and cementum and soft tissues such as gingiva and periodontal ligament. Left uncontrolled, periodontitis can eventually lead to severe tooth loss and edentulous and masticatory dysfunction. Indeed, periodontitis is the main reason for tooth loss in adults [1].

Periodontitis is not just a local lesion limited to the oral cavity, but closely linked to systemic health [3]. For instance, data from the National Health and Nutrition Examination Survey in the United States shows that moderate and severe periodontitis enable dampening of lung function [14]. A recent assessment indicates that periodontitis may contribute to poor coronavirus disease 2019 (COVID-19)-related outcomes [15]. Even within ‘self-perceived healthy’ adults, existing severe periodontitis could well indicate the possible presence of multiple inflammatory comorbidities [16]. Moreover, periodontopathogens such as *Porphyromonas gingivalis* (*P. gingivalis*) is associated with cardiovascular disease [17,18,19], cancer [20,21,22], insulin resistance [23,24], Alzheimer’s disease [25,26], and adverse pregnancy outcomes [27,28]. Furthermore, clinical periodontal treatment is able to improve the anemic status [29], modulates endotoxemia and stool microbial dysbiosis [30], could reduce the risks of perinatal mortality and preterm birth [31], and is beneficial for the effective management of type 2 diabetes [32,33,34].

Periodontitis has become a huge socioeconomic burden worldwide [35]. According to the first Global Burden of Disease (GBD) Study, severe periodontitis affects 11.2% of the entire global population and has been ranked as the sixth most prevalent disease in humans [4,36]. In China, periodontal disease could be detected among 90% of adults, and more than 30% of adults are suffering from severe periodontitis [37]. Notably, the global age-standardized prevalence rate of severe periodontitis increased by 8.44% during the last 30 years (from 1990 to 2019) [38]. Huge expenditure has been paid for direct periodontal treatment and indirect productivity losses. In 2010 alone, the indirect costs resulting from dental diseases were approximately $144 billion worldwide, among which 44% was due to severe tooth loss and 38% to severe periodontitis [4]. In 2018, around USD 154 billion and EUR 158 billion costs were caused by periodontal disease in the US and Europe respectively [39].

In summary, periodontitis as a ‘local’ lesion in the oral cavity can result in huge disasters to systemic health and to the entire world. Thus, more efforts are urgently needed for the prevention and treatment of periodontitis. To control periodontitis successfully, it is necessary to clarify the pathogenesis of periodontitis, which means the biological processes that lead to the disease.

### 2.2. Pathogenesis of Periodontitis: Infection and Inflammation

#### 2.2.1. Infection: Initiation of Periodontitis

Researchers in the earlier era proposed that calculus around the teeth was the local etiological factor of the disease. Following the expansion of our knowledge on microbiology, the importance of bacteria in the etiology began to rise. The last century has successively witnessed the occurrence of the ‘nonspecific plaque hypothesis’ (NSPH) [40], the ‘specific plaque hypothesis’ (SPH) [40], the ‘ecological plaque hypothesis’ (EPH) [41], and the ‘keystone pathogen hypothesis’ (KPH) [42,43]. The four hypotheses represent the development of our knowledge on the etiology of periodontal disease. In spite of some misunderstandings in earlier days, we are indeed gradually approaching the truth. Actually, the initiation of periodontal diseases is the combination of the NSPH, EPH, and KPH [44]. In the light of these theories, mechanical plaque removal is still the most widely applied method for disease prevention and treatment. Nevertheless, the development of periodontitis is a highly complex process, which is affected by both the pathogens and the host. Fully understanding how the host determines the development of periodontitis is of critical importance for preventing tissue damage caused by the disease.

#### 2.2.2. Inflammation: Progression of Periodontitis

In the 1990s, people began to realize that, although bacteria play essential role in the initiation of periodontitis, they are inadequate to cause severe hard-tissue destruction. A classical model of periodontal disease pathogenesis was developed by Page and Kornman in 1997, demonstrating that the microbial challenge and the host response influence each other, leading to the progression of periodontitis [45]. This model highlighted for the first time that tissue breakdown is not only caused by the direct effects of bacteria, but also results from the immune–inflammatory response. Moreover, the degree of periodontal tissue destruction varies greatly among individuals due to differences in the host’s genetic risk factors together with the environmental and acquired risk factors. Based on this model, simple removal of the microbial flora is insufficient for periodontal treatment—risk factors from the host and the environment need to be considered as well [46]. 

In 2015, a new model of periodontal disease pathogenesis [47] clarifies the concept of ‘clinical health’, meaning a symbiotic relationship between oral microorganisms and the host, but not a condition without any microbes. Indeed, a health-promoting biofilm is necessary and equally important for maintaining the symbiotic state. Moreover, unlike the classical paradigm stating a unidirectional route from the pathogenic microflora to inflammation, it is now clear that inflammation also facilitates the biofilm formation and function. Thus, further investigations are required to develop novel approaches for resolving the chronic inflammation lesion and re-establishing the symbiotic relationship between the oral flora and the host.

### 2.3. Periodontal Tissue Destruction: Consequences of Inflammation

Inflammation is a complex biological response of our body when facing harmful stimuli such as pathogens. During the process of inflammation, all kinds of immune cells, such as neutrophils, monocytes/macrophages, dendritic cells, and activated T-cells and B-cells, gather at the disease site. A cluster of (pro)inflammatory cytokines, enzymes, and mediators are secreted by them, accompanied with increased levels of ROS. These components form as a network fighting against the invading pathogens, while resulting in tissue destruction at the same time.

#### 2.3.1. Inflammation-Induced Destruction: Caused by Cytokines

**Figure 1:**
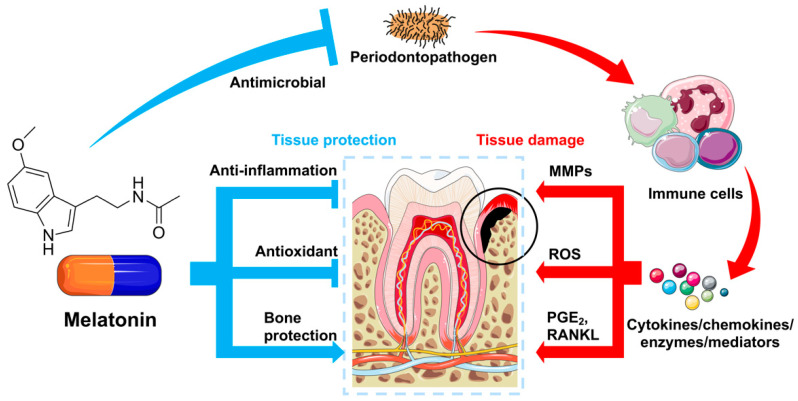
Melatonin exerts multitudinal biological functions for periodontal tissue protection. When facing challenges from periodontopathogens, immune cells secret a cluster of (pro)inflammatory cytokines, chemokines, enzymes, and mediators, which cause tissue damage through various mechanisms (see the text above for details). Melatonin protects periodontal tissues from destruction via its antimicrobial, anti-inflammation, antioxidation, and bone protection effects (see the text below for details).

Increased serum levels of cytokines and mediators, such as IL-1, IL-6, IL-12, tumor necrosis factor-alpha (TNF-α), prostaglandin E_2_ (PGE_2_), and C-reactive protein (CRP), have been reported in patients with severe periodontitis [48]. These cytokines, on one hand, can damage periodontal tissues directly, leading to irreversible periodontal attachment loss [48]. On the other hand, cytokines act as key modulators of cellular responses by inducing intracellular signaling and modifying gene expression during periodontal inflammation. For example, IL-1 and TNF-α as proinflammatory cytokines upregulate the immune–inflammatory level and enhance the expression of many components including matrix metalloproteinase (MMP) and receptor activator of nuclear factor kappa-Β ligand (RANKL) [49]. These components in turn cause tissue damage through various mechanisms (Figure 1).

MMPs are a cluster of extracellular proteinases that exert multifunctions during various physiological events such as immune response and tissue repair. MMPs have potent ability to degrade extracellular matrix proteins; thus, their activation is tightly regulated such as by tissue inhibitors of metalloproteinases (TIMPs) [50] and by extracellular matrix metalloproteinase inducer (EMMPRIN) [51]. When uncontrolled inflammation like periodontitis occurs, the established MMP proteolytic cascades result in widespread periodontal tissue destruction [52]. Our group has identified higher expression of EMMPRIN in inflamed human gingiva than in healthy individuals [53]. Enhanced MMP-1, MMP-2, MMP-3, MMP-7, MMP-8, MMP-13, and MMP-9 levels have been detected in the gingival crevicular fluid (GCF) and saliva samples in periodontitis patients, in parallel with decreased periodontal parameters [54,55,56,57,58,59,60]. Moreover, active MMPs can modulate the biological functions of certain cytokines and chemokines as well, thus in turn regulate periodontal inflammation [52]. Herein, MMPs have been regarded as key regulators involved in periodontal tissue destruction and potential targets for periodontal treatment.

RANKL is a type II membrane protein that binds to RANK on osteoclast surfaces and functions as a key factor for osteoclast differentiation and activation. Osteoprotegerin (OPG) functions as a decoy receptor for RANKL, thus inhibiting osteoclastogenesis and bone resorption. The RANKL/OPG ratio in periodontal tissues determines the occurrence and degree of bone destruction, and enhancing expression of RANKL in periodontium is highly associated with bone resorption [61]. In periodontitis tissues, many more T cells and B cells express RANKL, as compared with healthy gingival tissues [62,63], thus leading to more bone resorption. Nevertheless, conventional periodontal treatment such as mechanical plaque removal cannot affect the RANKL/OPG ratio. So, novel approaches that could reverse the RANKL/OPG ratio might be an alternative choice for preventing bone destruction.

#### 2.3.2. Inflammation-Induced Destruction: Caused by ROS

Increasing evidence has identified the role of ROS in the pathogenesis of periodontitis during the past few years. Enhanced levels of oxidative stress markers and decreased total antioxidant status (TAS) have been reported in saliva, GCF, and plasma of periodontitis patients, in parallel with poorer clinical periodontal parameters and higher levels of oxidant-induced DNA damage, with reference to periodontally healthy controls [64,65,66]. Moreover, systemic disorders that have tight connections with periodontitis (e.g., type 2 diabetes, obesity, and rheumatoid arthritis) and unhealthy lifestyles could increase the production of ROS, which further worsens the periodontal condition [67]. Furthermore, periodontal treatment had beneficial effects on periodontal parameters and the levels of the oxidative stress markers and antioxidant status [64,68]. Herein, oxidative stress could function as a therapeutic target for periodontitis management. Indeed, numerous endeavors have been made to manage periodontitis using ROS scavengers and obtained promising results [69,70].

The excessive ROS is normally released by the ‘hyperactivated’ polymorphonuclear neutrophils (PMNs) under inflammatory condition [71]. PMNs are the most abundant white blood cells in humans and the first immune cell line of defense against periodontopathogens [72]. They play an essential role in maintaining periodontal health through phagocytosis and ROS production. Moderate levels of ROS help to eradicate invading pathogenic microbes and exert essential functions on immune regulation [73]. Whereas, an overabundance of ROS results in increased oxidant stress as well as reduced antioxidant capacity, which then lead to pathological alteration and eventually host tissue destruction [74]. 

Several mechanisms exist behind the periodontal tissue damage caused by the superfluous ROS. ROS-induced oxidative stress can directly damage lipid, nucleic acid, and protein, leading to lipid peroxidation, chromosome disruption, and protein denaturation. Moreover, ROS as a signaling molecule is able to regulate several inflammatory processes such as NF-κB signaling activation [75], NLRP3-induced inflammasomes assembling [76], and RANKL-stimulated osteoclastogenesis [77], which lead to cytokine-induced tissue damage, pyroptosis, and bone resorption, respectively. Furthermore, ROS is able to activate the key MMPs in periodontal tissues via direct enzyme oxidation [78], and the activated MMPs degrade extracellular matrix proteins, as discussed above. 

### 2.4. Current Concepts in Periodontitis Treatment and Further Perspectives

Recently, the European Federation of Periodontology (EFP) published two guidelines for the treatment of stage I to IV periodontitis, which might be the latest and most effective evidence-based approach for the management of periodontitis [79,80]. For stages I, II, and III periodontitis, a pre-established stepwise approach is recommended depending on the stage of the disease. Four steps are included in this guidance: (1) patients’ behavioral changes; (2) supra- and subgingival instrumentation (i.e., scaling and root planing); (3) proper periodontal surgical interventions; (4) regular supportive periodontal care [79]. Stage IV periodontitis is much more complex than stages I–III periodontitis; hence, a combined periodontal therapy involving different departments is needed. Additionally, compared with stages I–III periodontitis, patients with stages IV periodontitis should be more aware of their condition and give more attention to self-performed plaque control and risk factor control [80]. 

In general, the two guidelines emphasize the importance of subgingival dental biofilm control. All of these approaches are based on the etiology of periodontitis that periodontopathogens initiate the disease; hence, removal of all the subgingival dental biofilm is the most effective method and has to be the priority during periodontal treatment. However, even with similar quantity of dental plaque, the progress rate of periodontitis varies greatly among different people. As mentioned above, periodontal tissue destruction is caused by the inflammation-related cytokines and mediators, and the host’s genetic risk factors together with the environmental and acquired risk factors determine the degree of tissue damage. In this respect, the term ‘host-modulation therapy’ was introduced by Maria E. Ryan and Lorne M. Golub [81,82,83] and developed rapidly for the treatment of periodontitis.

Initially, anti-inflammation drugs such as inhibitors of PGE_2_ and cyclooxygenase (COX), or TNF-α antagonists were used as adjunctive host-modulation therapies, while none of them have been approved for clinical use due to their serious side effects [84]. Based on its potent antimatrix metalloproteinase ability [85,86], doxycycline (nonantibiotic formulations) have been approved for periodontitis treatment in the US, Canada, and Europe, and exert powerful effects on preventing tissue destruction [87]. Yet, considering the severe consequence, the high occurrence rate, and the relapse/recalcitrance of periodontitis, developing more host-modulation agents for periodontal treatment is still highly required. Concerning the pathogenesis of periodontitis, the newly developed agent should have the ability of anti-infection, inflammation regulation, antioxidation, and bone regeneration. In this regard, melatonin might be the most appropriate candidate. 

## 3. Melatonin: Biological Functions and Beneficial Effects on Periodontal Health

Melatonin (N-acetyl-5-methoxy-tryptamine) is an endogenous hormone exhibiting a broad spectrum of biological effects. It was initially isolated from bovine pineal glands [88], and many investigations have been made since then for this amazing molecule. Melatonin is mainly produced and released by the pineal gland, and is synthesized by other extrapineal tissues as well, such as heart, liver, placenta, kidney, gut, and bone marrow [89,90]. The biosynthesis and secretion pattern of melatonin and its application to cancer treatment have been reviewed by us recently [91]. So, the current review mainly focuses on the latest evidence for the protective role of melatonin in the pathogenesis of periodontitis, and the biological functions of melatonin that may be involved in periodontal treatment. 

### 3.1. Melatonin and Periodontal Health: Increasing Evidence

#### 3.1.1. Evidence from Clinical Observations

Numerous clinical trials have been performed to measure the melatonin levels in the samples from the oral cavity (e.g., plasma, saliva, GCF, and gingival tissue) of humans with and without periodontitis [92,93,94,95]. Recently, a systematic review and meta-analysis including 14 articles was performed and concluded that, compared with healthy controls, patients with chronic periodontitis exhibited a significantly lower level of melatonin in saliva [96] (Table 1). However, the included studies had limited quality and low level of evidence. More investigations with an increased sample size and stringent age and sex matching are required to obtain a convincing conclusion. Moreover, all of these results were obtained from cross-sectional studies; it would be more persuasive to perform longitudinal studies recording melatonin levels within the same individuals at a different stage of periodontal condition (i.e., from periodontal health to disease). Nevertheless, this evidence does make clear the potential effects of melatonin for maintaining periodontal health. 

#### 3.1.2. Evidence from Randomized Controlled Clinical Trials

Increasing evidence from clinical trials has proved that melatonin exhibits beneficial effects for the treatment of periodontitis. For instance, local delivery of melatonin gel as an adjunct to nonsurgical periodontal therapy (NSPT) helps to provide better clinical and radiographic outcomes [97,98]. Systemic administration of melatonin after one-stage full mouth NSPT results in a greater clinical attachment level (CAL) gain and probing depth (PD) reduction, with reference to NSPT with placebo treatment [99,100]. Those reported have been analyzed recently by several systematic review and meta-analyses, concluding that adjunctive melatonin supplementation (topical and systemic) can significantly improve the PD, CAL, and other key periodontal parameters [13,101]. Moreover, for those patients with type 2 diabetes, systemic application of melatonin was able to benefit their periodontal status [102] and ameliorate their inflammatory and antioxidant parameters [103,104]. Furthermore, in the field of implant dentistry, melatonin may exert positive effects on bone formation around implants [105,106,107], despite that the available data are still limited and further trials are required to support the clinical significance (Table 1). 

Despite the above evidence proving the beneficial effects of melatonin on periodontal treatment, several reports also showed no statistical difference for the improvement of periodontal parameters with or without melatonin treatment. For instance, Konecna and colleagues demonstrated that systemic administration of melatonin neither prevent alveolar bone loss nor reduce salivary markers of oxidative stress within a periodontitis rat model, and mouth rinse with melatonin did not demonstrate positive effects in patients with periodontitis [108]. Moreover, Faramarzi et al. revealed that, although melatonin reduced more serum ferritin levels than the control group, no statistical difference was calculated [109]. The occurrence of these discrepancies may due to the limited sample size, the low dosage of melatonin applied, or the short duration of the study. Hence, more long-term observations with larger sample size and appropriate drug concentration are needed for further confirmation.

### 3.2. Melatonin and Periodontal Health: Underlying Mechanisms 

As discussed above, periodontitis is initiated by bacteria-induced infection, yet the periodontal tissue destruction is mainly caused by the excessive immune–inflammatory response. The action of inflammation includes upregulated expressions of cytokines and chemokines (e.g., IL-1β, TNF-α, and MMPs), increased ROS levels, high RANKL/OPG ratio, and so on. Herein, in order to manage periodontitis and reduce the tissue damage it causes, elimination of the periodontopathogen-induced infection is required to reduce inflammatory cytokine expression levels, to control the ROS level in periodontal tissues, and to re-establish balanced bone metabolism. Notably, melatonin exerts multitudinal biological functions that are suitable for periodontal treatment (Figure 1); detailed mechanisms are described as follows.

#### 3.2.1. Antimicrobial Effects of Melatonin

Melatonin is an endogenous hormone that exhibits potent anti-infection ability as well [110]. In vitro studies have demonstrated that melatonin was able to inhibit the growth of *Pseudomonas aeruginosa*, *Acinetobacter baumannii,* and Methicillin-resistant *Staphylococcus aureus* [111]. The in vivo antibacterial action of melatonin is normally associated with immune responses, such as reducing inflammatory cytokine production [112] and accelerating healing from bacteria-induced damage [113]. However, only very few studies investigate the antimicrobial activity of melatonin against periodontopathogens. To give an instance, both melatonin and its receptor agonist ramelteon exhibit antimicrobial effects against planktonic-cultured *P. gingivalis*. Notably, they inhibit the formation of *P. gingivalis* biofilm, disrupt the established biofilm, and reduce the viability of *P. gingivalis* biofilm [114]. Considering the powerful antibacterial ability of melatonin, it is reasonable to suppose similar anti-infection effects of melatonin on oral pathogens such as *Tannerella forsythia* (*T. forsythia*) and *Aggregatibacter actinomycetemcomitans* (*A. actinomycetemcomitans*). Moreover, since the formation of persisters is one of the survival strategies for *P. gingivalis* [115,116], it would be promising to explore the antipersister potential of melatonin. Therefore, more investigations are needed to prove these hypotheses.

#### 3.2.2. Anti-Inflammation Effects of Melatonin

Since most periodontal destruction is caused by the abundant inflammatory responses, better control of inflammation may prevent tissue damage to a great extent. The current widely used anti-inflammation drugs such as aspirin, non-steroidal anti-inflammatory drugs (NSAIDs), and corticosteroids always lead to serious side effects such as gastrointestinal discomforts [117] and bone comorbidities [118]. Melatonin as a hormone has been proved to exert strong anti-inflammation effects with very few side effects [119]. Herein, investigations have been performed to verify whether melatonin could prevent periodontal tissue damage via controlling the inflammatory responses. For instance, Kara et al. have proved that in periodontitis-induced rats, melatonin reduced inflammatory cytokines (IL-1β and TNF-α) and minimized periodontal tissue destruction [120]. Moreover, periodontitis-induced rats exhibit high RANKL/OPG ratio, enhanced TLR4/MyD88 activity, and upregulated proinflammatory cytokine levels. Notably, melatonin remarkably normalizes RANKL/OPG signaling by depressing TLR4/MyD88-mediated proinflammatory cytokine production [121]. Furthermore, IL-1β-induced CXCL-10, MMP-1, and TIMP-1 production in human periodontal ligament cells could be decreased by melatonin as well [122].

#### 3.2.3. Antioxidant Effects of Melatonin 

It is speculated that the origination of melatonin can date back to 3.0–2.5 billion years ago, when melatonin was designed to neutralize the toxic O_2_ in photosynthetic bacteria during photosynthesis. After almost 3 billion years evolution, the functions of melatonin have expanded greatly while the original antioxidant function has been maintained [123]. Nowadays, it is widely acknowledged that melatonin is a potent free radical scavenger and antioxidant. Different from other classical antioxidants, the metabolites of melatonin are able to neutralize oxygen derivatives as well. Thus, the cascade reaction makes melatonin much more powerful than other antioxidants such as vitamin C, vitamin E, glutathione, and NADH [124]. 

Owing to increasing attention on ROS for its tissue damage effects, numerous efforts have been made to control excessive ROS in periodontal tissue. As the most potent antioxidant substance, melatonin might be an excellent candidate. A randomized controlled clinical trial showed that melatonin significantly enhanced the antioxidative capacity (TAC) and inhibited the MMP-9 levels in GCF [98]. A meta-analysis of two RCTs revealed that in periodontitis patients with diabetes, combined NSPT with melatonin remarkably reduce the periodontal pocket depths, with reference with NSPT alone [125]. In gingival fibroblast from Wistar rats, glutamate (GLUT) and DL-buthionine sulfoximine (BSO) treatment lead to the production of superoxide anion and cell apoptosis, which can be totally counteracted by melatonin [10]. In periodontitis-induced rats, melatonin alleviates the oxidative stress caused by periodontal inflammation by inhibiting the inflammatory cytokine expression and restoring the antioxidant concentration [120]. Moreover, *P. gingivalis* has been proved to elevate oxidative stress and inflammatory response in human aortic endothelial cells via the NF-κB-BMAL1-NF-κB signaling loop, thus accelerating atherosclerosis progression. Notably, melatonin combined with metronidazole reversed the superoxide radical production and proinflammatory cytokines elevated by *P. gingivalis*. Thus, the combination of metronidazole and melatonin might be an alternative approach for atherosclerotic cardiovascular diseases [126]. 

#### 3.2.4. Bone Protection Effects of Melatonin

Bone resorption and tooth loss are the most serious consequence of periodontitis. How to prevent bone damage and re-establish the balanced bone metabolism is the primary objective during periodontal treatment. Through various mechanisms, melatonin has been demonstrated to exert beneficial potential on bone regeneration. As an illustration, the proliferation and synthesis rate of type I collagen are stimulated by melatonin in human bone cells and the human osteoblastic cell line [127]. Moreover, melatonin promotes osteogenesis in MC3T3-E1 cells by activating Sirtuin 1 [128], promotes bone marrow mesenchymal stem cell osteogenic differentiation [129], and inhibits adipogenesis yet enhances osteogenesis of human mesenchymal stem cells [130]. Furthermore, melatonin prevents bone resorption via attenuating RANKL-induced osteoclastogenesis [131,132]. 

Melatonin could protect the bone in the oral cavity as well. In rats with experimental periapical lesions, melatonin exerts anti-inflammatory and bone-protection activities by inhibiting IL-1β, RANK, and RANKL expression levels while enhancing OPG expression level. Moreover, melatonin significantly decreases the bacteria localization scores in periodontal tissues [133]. The osteogenic differentiation of dental pulp mesenchymal stem cells (DPSCs) can be enhanced by melatonin and in vivo bone defects can be rescued by melatonin-preconditioned DPSCs [134,135]. In rats with experimental periodontitis, melatonin treatment decreases serum terminal C telopeptide of collagen Type I levels and increases bone alkaline phosphatase levels. Alveolar bone resorption, myeloperoxidase activity, and RANKL and osteoclast activity are statistically downregulated by melatonin [136]. Melatonin could also protect drug-induced damage in osteoblasts. For instance, the application of chlorhexidine results in poor morphology of MC3T3 cells, leads to the upregulation of total ROS and superoxide levels in the cells, and diminishes the number of vital and metabolic active osteoblasts. Notably, melatonin is able to alleviate these damages caused by chlorhexidine in MC3T3 cells, and thus protects osteoblasts during chlorhexidine treatment [137]. For the prevention of peri-implantitis, melatonin could be a potent agent as well. In the lipopolysaccharides (LPS)-induced peri-implantitis rat model, melatonin dampens the proinflammatory cytokine expression, decreases the osteoclast numbers, prevents alveolar bone damage, and reduces the peri-implantitis incidence. The osteoclastic formation and function are suppressed, and the osteoblastic differentiation and function are promoted by melatonin in vitro as well [11]. 

#### 3.2.5. Other Effects of Melatonin 

Apart from the biological functions above, melatonin exhibits protective effects on oral tissues under harmful conditions as well. For instance, melatonin administration is able to decrease the oxidative stress and protect periodontal tissues caused by radiation therapy [138]. Moreover, melatonin attenuates the senescence of human periodontal ligament cells (PDLSCs) caused by ethanol-stimulation [139] and long-term ex vivo culture [140]. 

In summary, increasing evidence has identified the beneficial effects of melatonin on maintaining periodontal health and on periodontal treatment. The multiple biological functions of melatonin facilitate its protective role in periodontal tissues. Whereas most evidence comes from laboratory work, there is still a long way to go before the widespread administration of melatonin as an adjunctive therapy for periodontal treatment. More investigations are needed to determine the dosage and delivery approach for melatonin during its application. 

## 4. Melatonin and Periodontitis-Related Systemic Diseases: Far More Than Oral Cavity

As mentioned above, periodontitis is a ‘local’ lesion in the oral cavity yet is involved in the development of various systemic comorbidities. At the same time, systemic diseases and disorders could affect the development and consequence of periodontitis as well. So, in this part, we describe how melatonin favors those periodontitis-related systemic comorbidities. 

### 4.1. Melatonin and Diabetes Mellitus

The relationship between diabetes mellitus and periodontitis has drawn people’s attention worldwide for many years. It has been long since diabetes was considered as a major risk factor of periodontitis [141]. Indeed, periodontitis has been regarded as the sixth complication of diabetes ever since the 1990s [142]. At the same time, periodontitis has a negative effect on glycemic control [143], and periodontitis patients have higher prevalence of type 2 diabetes [144]. Additionally, periodontal treatment contributes to better glycemic control within type 2 diabetes patients [33,34]. This bidirectional interrelationship between diabetes and periodontal diseases inspire us to treat them together with the cooperation of different departments. 

Diabetes is characterized by high blood glucose levels and glucose intolerance, together with lipid and carbohydrate metabolic disorders [145]. These disturbances always cause inflammatory changes in the body, including enhanced RANKL/OPG ratio, increased proinflammatory mediator expression, and abundant ROS production [146]. Additionally, periodontitis-related tissue destruction is caused by too much ROS and an abnormal RANKL/OPG ratio. Herein, better control of diabetes could facilitate the treatment outcome of periodontitis. In vivo studies have proved that melatonin is able to decrease osteoclastic activity and reduce hyperglycemia-induced oxidative stress and alveolar bone loss in rats with diabetes and periodontitis [145,147]. Clinical trials have identified the moderating effects of melatonin on salivary RANKL/OPG ratio [148] as well as the reduction in salivary acid phosphatase, alkaline phosphatase, osteopontin, and osteocalcin concentration in patients with diabetes and periodontal disease [149]. Moreover, systemic administration or topical application of melatonin alleviates the inflammatory condition and improves periodontal status in diabetes patients with periodontitis [102,103,150]. Periodontal pocket depths were significantly reduced when combining melatonin with NSPT in periodontitis patients with diabetes [125]. 

Apart from the benefits to the local periodontal parameters, melatonin also favors the systemic conditions of diabetes patients with periodontitis. For instance, individuals with diabetes and periodontal disease may present high levels of serum C-reactive protein and IL-6, which could be decreased by local application of melatonin [151]. When combined with NSPT, melatonin leads to better glycemic control in periodontitis patients with type 2 diabetes [12,102]. In pinealectomyzed rats with periodontal disease, systemic administration of melatonin could prevent insulin resistance and increase plasma insulin levels [152]. Rats with apical periodontitis exhibit low insulin sensitivity and impaired insulin signaling, which could be rescued by melatonin [153]. 

### 4.2. Melatonin and Cardiovascular Diseases

The increased risk of periodontitis on cardiovascular diseases has been widely investigated. Periodontitis patients have a higher prevalence of cerebrovascular disease (CVD), and periodontal treatment produces a reduction in the incidence of CVD events [154,155]. It is postulated that the bacteremia caused by periodontitis results in bacterial invasion of endothelial cells, and this has been proved by the fact that specific oral bacterial species have been detected in cardiovascular specimens [156]. 

Only several studies explore how melatonin affects periodontitis-induced cardiovascular damage. For instance, melatonin combined with metronidazole reversed the superoxide radical production and proinflammatory cytokines elevated by *P. gingivalis* in human aortic endothelial cells. Thus, the combination of metronidazole and melatonin might be an alternative approach for atherosclerotic cardiovascular diseases [126]. Moreover, the expression levels of malondialdehyde (MDA), MMP-9, and cardiac Troponin-T (cTnT) in cardiac left ventricular tissue were upregulated in experimental periodontitis rats, and could be downregulated remarkably by melatonin [157]. Although no obvious antioxidant effects of melatonin were detected in this trial, another in vivo study demonstrated higher glutathione peroxidase level in periodontitis + melatonin group than periodontitis + saline solution group [158]. Thus, potential protective effects of melatonin on cardiovascular tissues might exist, but more investigations are required to support this conclusion.

### 4.3. Melatonin and Kidney Disease

The association between kidney disease and periodontitis has been discussed for the past few years [159]. On one hand, the impaired immune system in patients with kidney disease leads to high risks of infectious diseases such as periodontitis. On the other hand, periodontal pathogens and their virulence factors such as LPS, fimbriae, and gingipains could transfer from periodontal lesions to the kidney by the bloodstream, and periodontitis-induced inflammatory cytokines cause kidney damage as well [160]. 

Very few studies investigate the role of melatonin in kidney damage within those periodontitis patients. It has been revealed that in LPS-induced periodontitis rats, the increased serum aspartate aminotransferase, alanine transaminase, and urea nitrogen levels could be ameliorated with melatonin treatment [161]. A recent study demonstrated that periodontitis enhanced the levels of proinflammatory cytokines (TNF-α and IL-1β), oxidative stress (MDA), and proteases (MMP-8, MMP-9, and cathepsin D) in rat kidneys, while melatonin suppressed them significantly. Nevertheless, melatonin failed to rescue the impaired renal function [162]. More investigations are needed to further explore the connection between periodontal treatment and kidney disease and how melatonin affects this process. 

### 4.4. Melatonin and Obesity

It has been concluded that overweight or obese individuals have a higher risk of periodontitis. Bone marrow adiposity leads to decreased osteoblasts and increased osteoclastogenesis [163]. Excessive white adipose tissue results in enhanced ROS and inflammatory cytokines production, which in turn causes periodontal tissue damage [164]. Periodontitis patients with obese harbored higher levels of periodontopathogens such as *A. actinomycetemcomitans*, *T. forsythia,* and *Fusobacterium nucleatum* [165]. On the other hand, periodontitis could increase the risk of obesity as well [164]. Periodontitis-related insulin resistance results in hyperinsulinemia [23], which further promotes obesity [166]. Periodontitis-induced masticatory dysfunction forces patients to select a soft, high-fat/high-calorie diet, which facilitates fat accumulation [167].

Only two studies from the same group investigate the connections between melatonin and periodontitis associated with obesity. In rats with comorbidities of obesity and periodontitis, plasma melatonin levels were significantly lower with reference to controls and to those rats with only obese or periodontitis [168]. Notably, adjunctive melatonin therapy with periodontal treatment in these experimental rats remarkably prevented alveolar bone loss and exerted protective anti-inflammatory effects. These effects were much better than the adjunctive usage of chlorhexidine [169]. Although melatonin supplementation has been proved to reduce body weight and prevent obesity-related complications in obese patients or mice [170,171], there are still no reports on whether melatonin could facilitate body weight control in periodontitis patients associated with obesity. Additional investigations are required to verify the beneficial effects of melatonin on periodontitis-related obesity. 

### 4.5. Melatonin and Coronavirus Disease 2019 (COVID-19) 

COVID-19 has spread globally and brought about huge disasters for almost the past three years. Many components of the established cytokine storm during COVID-19 are similar to the cytokine expression profile of periodontitis [172]. Thus, the possible influence of periodontitis on COVID-19 has been broadly discussed. It has been demonstrated that periodontitis patients had a higher risk of acquiring severe COVID-19 complications, death, ICU admissions, or assisted ventilation [15,173]. The underlying mechanisms have been identified as well [174]. For instance, angiotensin-converting enzyme 2 (ACE2), one of the key receptors for the invasion of SARS-CoV-2, is highly expressed on the epithelial cells of oral mucosa [175], and could be upregulated in patients with periodontal disease and diabetes [176]. Moreover, periodontopathogens aspirated into the lungs could facilitate more SARS-CoV-2 invasion and replication [177]. Therefore, better management of periodontitis may help to reduce infection and transmission of SARS-CoV-2. 

Increasing evidence has proved that melatonin as an adjunctive agent exhibited beneficial effects for COVID-19 prevention and treatment [178,179], although there is still no direct evidence supporting the possible role of melatonin in COVID-19 outcomes with periodontitis patients. It is hypothesized that melatonin may prevent the activation of NLRP3 inflammasome, thus protect tissue damage from COVID-19 and periodontitis [180]. Thus, more investigation from both laboratory work and clinical tests are still required to support the hypothesis.

Above all, periodontitis is closely related with multiple systemic diseases and disorders, and melatonin exhibits beneficial effects not only on periodontal health but also on general conditions (Table 2). Nevertheless, the current investigations are far from enough, more research is required to explore how melatonin facilitates the treatment of other periodontitis-related comorbidities such as Alzheimer’s disease, adverse pregnancy outcomes, and rheumatoid arthritis. Overall, based on the present evidence, the application of melatonin should be promising and harbors a bright future. 

## 5. Conclusions and Perspectives

Chronic infectious and inflammatory diseases have emerged as a major global health burden [181]. Periodontitis as a bacteria-induced, chronic infection/inflammatory disease destroys the periodontium and contributes to various systemic disorders. Based on our understanding of the pathogenesis of periodontitis, host-modulation therapy should be an adjunctive approach applied with classical SRP during periodontal treatment. Melatonin, a pleiotropic hormone that has been universally applied for treating sleep disorders, is justified as a host modulating agent during periodontal treatment, due to its anti-infection, anti-inflammation, antioxidant, and bone remodeling capacities. Increasing evidence from clinical practice and laboratory work has proved the beneficial effects of melatonin on periodontal health and general healthcare. However, it is still inadequate for our current knowledge of melatonin in the field of periodontology. There is a lack of standard guidelines for the clinical administration of melatonin in periodontal treatment. Although both short-term and long-term systemic usage of melatonin is safe, a few mild side effects such as dizziness, headache, nausea, and sleepiness may occur among some individuals [182]. Thus, local delivery might be more suitable for its oral application. Therefore, more investigations are needed to illustrate the proper dosage and precise delivery approaches of melatonin for periodontitis treatment. Considering the multiple beneficial effects of melatonin on human health, we do hope this review can help to enrich our understanding of the management of periodontitis and periodontitis-related systemic comorbidities. More host modulating agents besides melatonin would dramatically contribute to precisely and effectively tackling inflammatory disease-induced tissue damage. 

## Figures and Tables

**Table 1:** Increasing evidence for the beneficial role of melatonin in patients with periodontitis.

Study Type	Main Findings	References
Clinical observations	Salivary and GCF melatonin levels decrease in subjects with periodontitis	[92]
	Melatonin levels in both GCF and saliva were lower in patients with chronic periodontitis and aggressive periodontitis than in patients with gingivitis and in healthy subjects	[93]
	Melatonin levels were significantly lowered in gingival tissue samples of chronic periodontitis patients compared to healthy individuals	[94]
	Melatonin in GCF levels were significantly higher in the control than the GAgP and CP groups	[95]
	Patients with chronic periodontitis exhibited a significant lower level of melatonin in saliva, with reference to healthy controls	[96]
Randomized controlled clinical trials	Intrapocket application of 1% melatonin gel for 1 week combined with NSPT helps to get better clinical and radiographic outcomes	[97]
	Intrapocket application of 5% melatonin gel weekly once for 4 weeks combined with NSPT improves clinical and radiographic outcomes	[98]
	Taking melatonin capsules (1 mg per day for 1 month) after NSPT results in a greater CAL gain and PD reduction	[99]
	A 2-month regimen of 10 mg oral melatonin capsule once daily before bedtime after NSPT results in a greater CAL gain and PD reduction	[100]
	Adjunctive melatonin supplementation (topical and systemic) could significantly improve the PD, CAL, and other key periodontal parameters (systematic review and meta-analysis)	[13,101]
	Melatonin (tablets containing 6 mg of melatonin, once a day for 8 weeks) benefits periodontal status of type 2 diabetes patients	[102]
	Melatonin (tablets containing 6 mg of melatonin, once a day for 8 weeks) ameliorates the inflammatory and antioxidant parameters of type 2 diabetes patients	[103,104]
	Melatonin exerts positive effects on bone formation around implants (systematic review and meta-analysis)	[105,106]
	The application of ABG/melatonin (VIVAMAX3; AMOUN Pharmaceutical Industries Co. (APIC), Cairo, Egypt) exerts positive effects on bone formation around implants	[107]

Abbreviations: GCF, gingival crevicular fluid; GAgP, generalized aggressive periodontitis; CP, chronic periodontitis; NSPT, nonsurgical periodontal therapy; CAL, clinical attachment level; PD, probing depth; ABG, autogenous bone graft.

**Table 2:** Melatonin and periodontitis-related systemic diseases.

Periodontitis-Related Systemic Diseases	Function Mechanisms of Melatonin	Application Methods of Melatonin	Reference
Diabetes mellitus	Decreasing osteoclastic activity; reducing hyperglycemia-induced oxidative stress and alveolar bone loss	Male Wistar rats, intraperitoneal injection of 10 mg/kg/day for 4 weeks	[145]
		Male Sprague Dawley rats, 10 mg/body weight intraperitoneal dose of melatonin once a day for 14 days	[147]
	Moderating salivary RANKL/OPG ratio	Diabetic patients, topical application of melatonin (1% orabase cream formula) once daily for 20 days	[148]
	Reduction in salivary acid phosphatase, alkaline phosphatase, osteopontin, and osteocalcin concentration	Diabetic patients, topical application of melatonin (1% orabase cream formula) once daily for 20 days	[149]
	Ameliorating inflammation; improving periodontal status	Diabetic patients, tablets containing 6 mg of melatonin, once a day for 8 weeks, 1 h before bedtime	[102]
		● Diabetic patients, tablets containing 6 mg of melatonin, once a day for 8 weeks, 1 h before bedtime	[103]
		Diabetic patients, topical application of melatonin (1% orabase cream formula) once daily for 20 days	[150]
	Reducing periodontal pocket depths	Systematic review and meta-analysis	[125]
	Reducing serum C-reactive protein and IL-6	Diabetic patients, topical application of melatonin (1% orabase cream formula) once daily for 20 days	[151]
	Leading to better glycemic control combined with NSPT	● Diabetic patients, tablets containing 6 mg of melatonin, once a day for 8 weeks, 1 h before bedtime	[12]
	Preventing insulin resistance; increasing plasma insulin levels	Male Wistar albino rats, 5 mg/kg body weight in drinking water for 28 days	[152]
	Improving insulin sensitivity; rescuing impaired insulin signaling	Male Wistar rats, 5 mg kg^−1^ melatonin (diluted in drinking water) for 60 days	[153]
Cardiovascular diseases	Reversing the superoxide radical production and proinflammatory cytokines when combined with metronidazole	C57BL/6J mouse, 5 mg/kg intraperitoneal dose of melatonin once a day for 16 weeks	[126]
	Downregulating MDA, MMP-9, and cTnT expression levels	Male Sprague-Dawley rats, 10 mg/body weight intraperitoneal dose of melatonin once a day for 14 days	[157]
	Improving glutathione peroxidase level	● Wistar Albino male rats, intraperitoneal injection of 10 mg/kg/day for 2 weeks	[158]
Kidney diseases	Downregulating serum aspartate aminotransferase, alanine transaminase, and urea nitrogen levels	Female Wistar albino rats, intraperitoneal injection 50 mg/kg of melatonin, daily for 10 d	[161]
	Suppressing expression levels of proinflammatory cytokines (TNF-α and IL-1β), oxidative stress (MDA and OSI), and proteases (MMP-8, MMP-9, and CtD)	Male Sprague Dawley rats, daily intraperitoneal dose of 10 mg/kg of melatonin	[162]
Obesity	● Preventing alveolar bone loss and exerting protective anti-inflammatory effects	Wistar rats, 25 μg/mL of melatonin dissolved in the drinking water for 4 weeks	[169]
COVID-19	Preventing the activation of NLRP3 inflammasome	● Hypothesis	[180]

Abbreviation: RANKL, receptor activator of nuclear factor kappa-Β ligand; OPG, osteoprotegerin; IL, interleukin; NSPT, nonsurgical periodontal therapy; MDA, malondialdehyde; MMP-9, matrix metalloproteinase-9; cTnT, cardiac Troponin-T; TNF, tumor necrosis factor-alpha; OSI, oxidative stress index; CtD, cathepsin D; NLRP3, NOD-like receptor thermal protein domain associated protein 3.

## Data Availability

Not applicable.
